# Reply: K-Ras: a prognostic factor for survival in non-small cell lung cancer

**DOI:** 10.1038/sj.bjc.6603113

**Published:** 2006-04-25

**Authors:** C Mascaux

**Affiliations:** 1 Institut Jules Bordet Departement de Medecine Interne et Laboratoire d'Investigation Clinique et d'Oncologie Experimentale HJ Tagnon Rue Heger-Bordet 1, Bruxelles B-1000, Belgium

**Sir,**

We would like to thank Gianluigi Ferreti and his colleagues for their interest in our systematic review.

In their letter, Ferreti *et al.* interrogated on the prognostic role of Ras in NSCLC. They cited seven studies with apparent conflicting results. Some comments have to be made. First, we discussed in our articles the controversial results of the literature relative to biological prognostic factors, justifying the need for meta-analyses, as we reported with K-Ras (Mascaux *et al*, 2005) and other biomarkers previously such as p53, bcl2, VEGF, Neu, Ki-67 or EGFR.

Secondly, we have some concerns with the studies cited by Ferreti *et al.* Some of these articles were not included in our systematic review for the following reasons:

- no prognostic analysis (Ferretti *et al*, 2000);
- no survival prognostic analysis but assessment of the predictive role of K-Ras for response to chemotherapy (Eberhard *et al*, 2005; Winton *et al*, 2005);
- assessment of K-Ras mutation on resected tumours after induction chemotherapy (Broermann *et al*, 2002);
- publication after the deadline for the selection of the studies to be included in the meta-analysis (Zhu *et al*, 2004).

The last two studies that Ferreti *et al* commented (Moldvay *et al*, 2000; Schiller *et al*, 2001) were included in our systematic review and were aggregated with the 26 other evaluable studies for meta-analysis.

In conclusion, our systematic review showed that K-Ras is a potential prognostic factor for survival in lung cancer according to the literature published on this topic till end of 2003. The predictive role of K-Ras for response to chemotherapy and the impact of K-Ras mutation on survival in patients receiving induction chemotherapy before surgery were not the subjects of our meta-analysis and need further investigations.
